# Background insect herbivory increases with local elevation but makes minor contribution to element cycling along natural gradients in the Subarctic

**DOI:** 10.1002/ece3.6803

**Published:** 2020-09-21

**Authors:** Jeppe A. Kristensen, Anders Michelsen, Daniel B. Metcalfe

**Affiliations:** ^1^ Department of Physical Geography and Ecosystem Science Lund University Lund Sweden; ^2^ Geological Survey of Denmark and Greenland Copenhagen Denmark; ^3^ Department of Biology Terrestrial Ecology Section University of Copenhagen Copenhagen Denmark; ^4^ Center for Permafrost University of Copenhagen Copenhagen Denmark; ^5^ Department of Ecology and Environmental Sciences Umeå Umeå Universitet Umeå Sweden

**Keywords:** carbon cycling, fast cycle versus slow cycle, insect herbivory, nutrient cycling, space‐for‐time substitution, Subarctic mountain birch forest

## Abstract

Herbivores can exert major controls over biogeochemical cycling. As invertebrates are highly sensitive to temperature shifts (ectothermal), the abundances of insects in high‐latitude systems, where climate warming is rapid, is expected to increase. In subarctic mountain birch forests, research has focussed on geometrid moth outbreaks, while the contribution of background insect herbivory (BIH) to elemental cycling is poorly constrained. In northern Sweden, we estimated BIH along 9 elevational gradients distributed across a gradient in regional elevation, temperature, and precipitation to allow evaluation of consistency in local versus regional variation. We converted foliar loss via BIH to fluxes of C, nitrogen (N), and phosphorus (P) from the birch canopy to the soil to compare with other relevant soil inputs of the same elements and assessed different abiotic and biotic drivers of the observed variability. We found that leaf area loss due to BIH was ~1.6% on average. This is comparable to estimates from tundra, but considerably lower than ecosystems at lower latitudes. The C, N, and P fluxes from canopy to soil associated with BIH were 1–2 orders of magnitude lower than the soil input from senesced litter and external nutrient sources such as biological N fixation, atmospheric deposition of N, and P weathering estimated from the literature. Despite the minor contribution to overall elemental cycling in subarctic birch forests, the higher quality and earlier timing of the input of herbivore deposits to soils compared to senesced litter may make this contribution disproportionally important for various ecosystem functions. BIH increased significantly with leaf N content as well as local elevation along each transect, yet showed no significant relationship with temperature or humidity, nor the commonly used temperature proxy, absolute elevation. The lack of consistency between the local and regional elevational trends calls for caution when using elevation gradients as climate proxies.

## Graphical abstract

Moth outbreaks constitute well‐described and conspicuous disturbances in Subarctic birch forests, but the moth contributions to carbon and nutrient cycling at background densities remain poorly constrained. We, for the first time, show that insect background insect herbivory increase with elevation from the valley bottom toward the treeline in Subarctic mountain birch forests. Despite making a subordinate contribution to overall elemental cycling, the higher decomposability and earlier timing of insect deposits relative to senesced leaf litter may make this flux disproportionately important for ecosystem functions.

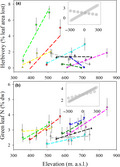

## 1 INTRODUCTION

Herbivores alter element cycling in terrestrial ecosystems (Bardgett & Wardle, 2003, 2010; Schmitz et al., 2018; Wardle et al., 2004). While less conspicuous than mammals, insect herbivores can exert a similar or even stronger control than mammals on ecosystem functioning (Hunter, 2001, Kristensen et al., 2020; Lovett et al., 2002; Risch et al., 2018; Silfver et al., 2020), particularly in forest ecosystems, where their impact is likely to intensify substantially with global change (Logan et al., 2003). Insect herbivory may cause early leaf abscission (Karban, 2007; Zvereva & Kozlov, 2014), leaf consumption (Galmán et al., 2018; Kozlov et al., 2015), alteration of aboveground versus belowground allocation of photosynthates (Ayres et al., 2004; Kristensen et al., 2020), and induction of plant defenses (Fürstenberg‐Hägg et al., 2013; Halitschke et al., 2008; Haukioja, 2005; Kessler et al., 2004). These effects alter the timing, quantities, and pathways of element fluxes, including the partitioning between fast‐ and slow‐cycle pathways through the decomposition foodweb (Frost & Hunter, 2004, 2007; Hunter et al., 2012; Kristensen et al., 2018).

Ecosystem studies of insect herbivory in high‐latitude forests have hitherto emphasized the importance of outbreaks (Jepsen et al., 2013; Sandén et al., 2020), especially as some species are increasing their ranges into new areas with climate warming (Jepsen et al., 2008, 2011). However, nonoutbreak, low‐intensity rates of insect herbivory, termed background insect herbivory (BIH), have attracted increasing attention in recent years (Kozlov & Zvereva, 2017). As BIH at high latitudes is generally low—1%–2% of the leaf area (Barrio et al., 2017) compared to the global average of ~8% (Kozlov, et al., 2015)—the rate will likely increase at high latitudes with global warming (Kozlov, et al., 2015), even if insect predation and parasitism also increase with temperature (Roslin et al., 2017; Virtanen & Neuvonen, 1999). It has been argued that BIH, despite the small annual contribution, may be more important for long‐term ecosystem functioning in a wide range of globally important ecosystems, including tropical (Metcalfe et al., 2014), temperate (Hunter et al., 2003), boreal (Metcalfe et al., 2016; Zvereva et al., 2012), and arctic systems (Barrio et al., 2017).

The fact that most of the global terrestrial organic C is stored at high latitudes (Hugelius et al., 2014), where global warming is most pronounced (ACIA, 2004), makes it particularly important to understand perturbations in this region. While low productivity ecosystems dominate at high latitudes (Higgins, Buitenwerf, & Moncrieff, 2016), subarctic birch forests constitute a relatively productive ecosystem (Sjögersten & Wookey, 2009). Therefore, perturbations in birch forests may exert disproportionately large impacts on regional biogeochemical cycling. Mountain birch (*B. pubescens*) provide the majority of the foliar biomass in the studied ecosystem (~65%, Dahlberg et al., 2004). Moreover, the mountain birch litter is more difficult to decompose than other types of detritus in these forests (Freschet et al., 2012), so herbivory on this species is particularly important for ecosystem level processes (Stark et al., 2007), partly through accelerating the recycling of resources in soils (Kristensen et al., 2018). The consequences of herbivory, by primarily the geometrid moths *Epirrita autumnata* and *Operophtera brumata,* have been demonstrated in terms of plant traits (Haukioja, 2003; Karlsson et al., 2004), plant (Jepsen et al., 2013; Sandén et al., 2020) and soil community composition (Kristensen et al., 2018; Parker et al., 2016; Saravesi et al., 2015), soil nutrient and carbon (C) turnover (Kaukonen et al., 2013; Kristensen et al., 2018; Parker et al., 2016; Sandén et al., 2020), and photosynthetic C‐fixation (Bjerke et al., 2014; Heliasz et al., 2011; Silfver et al., 2020). Nonetheless, quantification of one of the key mechanisms driving these changes—canopy‐to‐soil fluxes of C, N, and P through insect deposits—is still lacking. Elements channeled through insects are not a novel element input to the ecosystem, but rather an alternative pathway of transferring high‐quality organic matter from the canopy to the soil short‐circuiting the usual transfer of senesced litter by the end of the season. Most studies indicate that N is the main plant growth‐limiting nutrient in most forest systems (LeBauer & Treseder, 2008), but an increasing body of literature suggests that P is co‐limiting plant growth (Sundqvist et al., 2014; Vitousek et al., 2010). Further, nutrient limitation will become even more widespread in the future due to warming and CO_2_ fertilization, which may weaken the terrestrial ecosystem C‐sink (Fisher et al., 2012; Wieder et al., 2015). Therefore, it is relevant to assess the amounts of both N and P channeled through labile insect deposits (fast cycle) and recalcitrant litter (slow cycle), respectively. Insect deposits contain much larger amounts of nutrients compared to senesced litter (Kristensen et al., 2018) because insects feed on green leaves before the highly conservative subarctic birches resorb up to 60%–70% of their nutrients during senescence (Freschet et al., 2010; Nordell & Karlsson, 1995). Thus, the balance of elemental transfer between the litter and insect pathways regulate the timing and quality of soil substrate inputs, which can in turn lead to both increased and reduced soil C and nutrient turnover (Kristensen et al., 2018; Parker et al., 2016; Sandén et al., 2020).

N is also the growth‐limiting nutrient for the moths in our study system (Metcalfe et al., 2019). The foliar content of N is therefore expected to be an important driver of moth success, hence BIH level. In order to conserve nutrients from herbivores, host plants may increase the level of foliar chemical defense compounds (Fürstenberg‐Hägg et al., 2013; Haukioja, 2005). For example, the leaf content of bioactive specialized compounds, such as condensed tannins (CT), has been found to increase in leaves subjected to herbivory (Fürstenberg‐Hägg et al., 2013). Yet, the relationship between herbivory and plant defense compounds is not simple in natural systems. In fact, geometrid moth species in subarctic birch forests are rather tolerant to the birch chemical defense compounds (Haukioja, 2003, 2005). Therefore, it is only relevant for the birch to pursue this defense strategy when the growth‐limiting nutrient level in the leaves is so low that the insects have to eat large amounts of foliage to compensate for low nutrient concentrations (Haukioja, 2003). Thus, a negative relationship between the foliar content of the moth growth‐limiting nutrients and foliar defense compounds should be expected at the ecosystem scale.

Apart from the links to foliar chemistry, BIH also varies with climate, with an expected increase with warmer temperatures in high‐latitude systems (Barrio et al., 2017; Galmán et al., 2018; Kozlov, et al., 2015). Nonetheless, moth outbreaks most often appear close to the treeline in birch forests across subarctic Scandinavia rather than in valley bottoms, probably due to the higher likelihood of winter temperatures below their egg survival limit (<−35–37°C) along the valley bottoms. This is due to thermal inversion of air masses during winter (Hagen et al., 2007; Ruohomäki et al., 1997) and/or higher parasitism during the summer (Virtanen & Neuvonen, 1999). Yet, such decrease in top‐down controls on herbivory at higher local elevation may be confounded by decreasing bottom‐up controls. For example, the food quality (leaf N content) is also expected to increase with elevation (Körner, 1989; Read et al., 2014). Nonetheless, no systematic increase in BIH with the local elevation above the valley bottom has been found previously (Virtanen & Neuvonen, 1999), and such an increase would oppose the overall elevational decrease in herbivory at the global scale (Galmán et al., 2018).

In this study, we quantified BIH on the mountain birch (*Betula pubescens* var. *pumila*) along nine elevation gradients in Subarctic birch forests spanning a considerable portion of the natural climatic variation in the Fennoscandian mountain birch forests. We estimated the canopy‐to‐soil fluxes of C, N, and P per unit ground area in litter and insect deposits and compared them to other relevant sources of soil input of the same elements. We also evaluated the dependence of BIH and the resulting elemental fluxes on leaf chemistry, climate, elevation, and relative position over the valley bottom to identify potential abiotic and biotic drivers. Our setup with multiple gradients spanning a larger regional elevational gradient allowed us to test the universality of relationships between elevation and foliar loss to herbivory. We hypothesised that:

(H_1_) *Elemental fluxes*: The BIH levels in subarctic birch forests are minor compared to lower latitude systems, and consequently the annual contribution of nutrients to the soil through this channel is much smaller than other internal (i.e., recycling from litter) and external sources (i.e., fixation, deposition, weathering).

(H_2_) *Biotic controls*: The BIH level increases with concentration of leaf N, which is the growth‐limiting nutrient in the system. Consequently, we expected no strong relationship with leaf condensed tannin concentration, as this only plays a role as a defense compound at low N‐levels, where we already expected low foliar loss to herbivory. Moreover, we hypothesised that the importance of insect herbivores for channeling nutrients from the canopy to the soil would increase with stronger foliar nutrient resorption, as this would decrease the transfer of nutrients through senesced litter on an annual basis.

(H_3_) *Abiotic controls*: We expected a local scale increase in BIH with the relative position above the valley bottom along each transect toward the treeline, due to decreasing likelihood of extreme winter cold and summer parasitism, which are both most pronounced along valley bottoms. Yet, we also expected an overall BIH level increase with site temperature, as insect herbivory levels are generally higher in warmer mid‐latitude forests, but this effect may be substantially weakened by the expected local elevational increase in BIH.

## 2 MATERIALS AND METHODS

### 2.1 Study site and design

**Figure 1:**
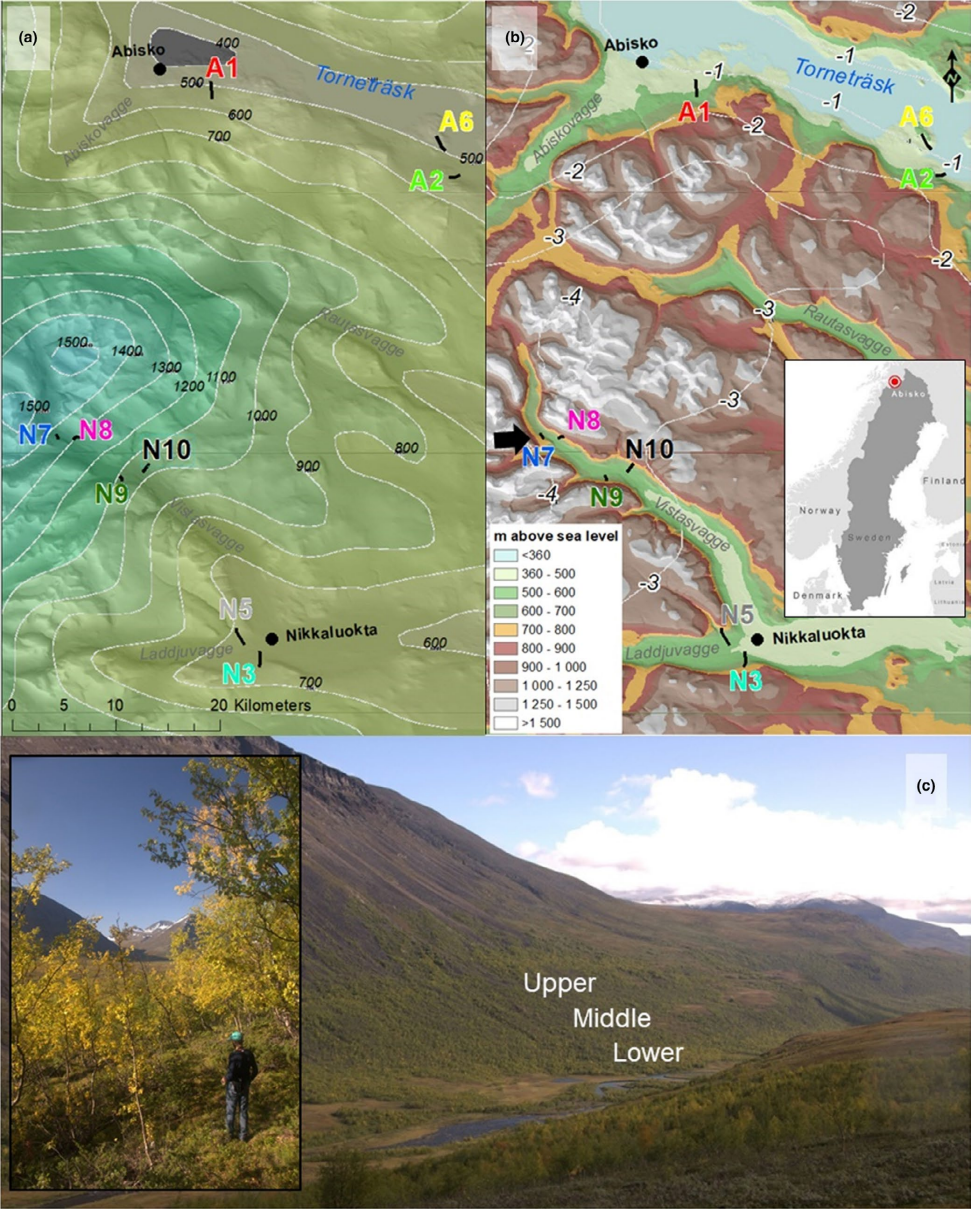
The study site and design. Mean annual precipitation (mm, background color in a) and temperature (°C, isolines in b) in the study area during the last normal period (1961–1990). The background color scale in panel b is a digital elevation model of the area. The short black lines in panels a and b show the location and names of the 9 transects. Colors of the transect names correspond to Figure 2. The red dot on the inset in panel b shows the location of the study area in Sweden. Panel c shows a south‐facing valley side in Vistasvagge, where transect N8 was located. The approximate relative positions of the three sites along this transect is shown in text. The black arrows in panel b show the location and direction of where the picture in panel c was taken. The inset in panel c shows a typical site, with a person for scale. Data: SMHI and Lantmäteriet. Photo credit: Thomas Heister

The study sites were in subarctic mountain birch forests near the Abisko Scientific Research Station, northern Sweden (68.35°N 18.82°E). All samples were taken in 2017. The layout was designed to capture as much of the regional climatic variation as possible within a relatively constrained geographic area (<50 km from Abisko) by taking advantage of the considerable climatic gradients created by the mountainous landscape between Abisko and the Kebnekaise complex (Figure 1). We established 9 elevation transects, each with a site near the valley bottom, one near the treeline, and one in the middle, making a total of 27 sites. Further, eight of the transects were paired as a north‐ and a south‐facing transect at four locations to control for the influence of aspect/solar radiation. The rationale behind this design was to assess the assumption underlying the use of elevation as a climate proxy: If there is a universal relationship between elevation and an ecological variable, the slope of the trendline in an X–Y plot would be similar at regional and local scale. To check the design, we included a plot of a commonly measured ecological variable, foliar N content, against elevation, as we expected this to show a clear and consistent increasing trend with elevation across scales (Körner, 1989), although potentially weaker at regional scale (Read et al., 2014).

According to a digital elevation model (DEM) from Lantmäteriet (50 m resolution), regional variation in elevation across the 27 sites is ~500 m (minimum 351 and maximum 845 m above sea level), while the minimum and maximum elevational difference along a single transect is 82 and 233 m, respectively. To control for aspect, we used the DEM to estimate the total annual solar radiation of 2017 for each site using the “Area Solar Radiation” function in ArcGIS 10.3 (ESRI, Redlands). Sites were preferentially established in dry locations with heath‐dominated ground vegetation; however, this was not possible in all instances, as some of the wetter transects were dominated by herbaceous ground vegetation (7 out of 27 sites with >50% herbs). The ground vegetation was dominated by typical Subarctic dry heath dwarf shrubs, particularly *Empetrum nigrum* ssp. *hermaphroditum* (Hagerup) Böcher*, Vaccinium myrtillus,* and *Vaccinium vitis‐idea*, with higher coverage of graminoids and forbs at the relatively wetter and more fertile sites and mosses and (rarely) lichens at the low fertility end (Table S1).

According to the Swedish Meteorological and Hydrological Institute Luftwebb record (4x4 km resolution) from the last normal period (1961–1990, isolines in Figure 1), the mean annual air temperature across our sites ranged between −2.5 ± 1.03 and −1.4 ± 1.03°C (mean ± *SE*), and the mean annual precipitation ranged from 404 ± 75 to 1,250 ± 158 mm. For the analyses below, we have used the average of the more recent period of 2000–2014 to derive meteorological variables more comparable to the study period. The mean annual soil temperature (averaged from summer 2016 to summer 2017) across all sites ranged between 1.7 ± 0.06 and 3.5 ± 0.1°C, while the mean annual growing season (averaged from 15/6 to 15/9 2017) soil temperature ranged between 6.7 ± 0.1 and 11.1 ± 0.2°C. These figures are slightly lower but comparable to the coarse scaled (4 × 4 km) modeled Luftweb record for the mean growing season air temperature per transect for the period 2000–2014 (mean: 10.7 ± 0.2°C, Table S1), so we believe the variance in mean growing season soil temperature reflects the site air temperature variance relatively well. Early growing season volumetric soil moisture content ranged from 11 ± 1.1 to 38 ± 2.5% across sites (Table S1). All sites were characterized by relatively acid (mean pH: 4.5 ± 0.06) and nutrient poor soils. Dissolved inorganic N content was low, and at the same level as dissolved P, while dissolved base cations (calcium, magnesium, sodium and potassium) were an order of magnitude more abundant than N and P (Table S1), suggesting no scarcity of other macronutrients than N. The bedrock geology was dominated by acidic bedrocks, and there was weak‐to‐moderate podzolisation at all sites. See the below section for methodology and Table S1 for full site characteristics.

### 2.2 Fieldwork and sampling

Measurements and samples were collected within a 20x20 m area at each site. After the *B. pubescens* leaves had fully expanded (late June), the leaf area index (m^2^ leaf m^−2^ ground, LAI) was estimated with the Hemisfer software, version 2.2 (Schleppi et al., 2007; Thimonier et al., 2010) based on ~10 hemispherical images per site (camera: Nikon Coolpix 4,500; lens: Nikon Fisheye converter fc‐e8 0.21x). In addition, we used ground slope correction (Schleppi et al., 2007), and the most recent algorithm for LAI estimation following manual thresholding (Gonsamo et al., 2018). We haphazardly selected ~30–50 fully expanded leaves from the lower canopy (~1.5–2.5 m above the ground) from a minimum of seven *B. pubescens* trees per site. Specific leaf area (m^2^/g dry mass, SLA) was estimated by scanning the fresh leaves to obtain the area, and then drying (40°C for 48 hr) and weighing. Annual *B. pubescens* leaf production (g dry mass m^−2^ yr^−1^, LP) per site was assumed to be equivalent to fully expanded canopy biomass in this deciduous species, which was estimated by dividing the LAI with the SLA (LP = LAI/SLA). After scanning and drying, the leaves were chemically analyzed (see below). In the late growing season (late August), ~30–50 freshly fallen or senesced yellow leaves still on the branches were collected from a minimum of 7 trees per site in order to determine leaf chemistry at senescence (see below). All leaves were dried (40°C for 48 hr) and ground before chemical analyses. In the late growing season, 4 large organic horizon samples (soil from ~1 m^2^ each sieved through 6 mm mesh) from each site were collected for a range of chemical analyses as indicators of site fertility (see below). Subsoil samples were collected in sampling rings (3 per site, ø: 5 cm, vol: 100 cm^3^, Eijkelkamp, Geisbeek, NL) and composited before analyses.

The insect herbivory level was estimated using a modified version of the method described by Crutsinger et al. (2008). At each site, we randomly selected 7 trees in the late growing season (August/September), after the vast majority of insect herbivory had terminated. Trees were selected from a distance (~15 m) where insect herbivory could not be detected, to minimize sampling bias (Zvereva & Kozlov, 2019). On each tree, 3 branches were haphazardly selected on which we visually estimated the percentage of leaf area lost to herbivory (0%, 0.01%–1%, 1%–5%, 5%–10%, 10%–20%, 20%–30% … 90%–100%) in the lower canopy (~1.5–2.5 m above the ground). Ten leaves were surveyed per branch, starting from the first full‐sized leaf and down the branch surveying every other leaf. When calculating the average herbivory level for each site, each observation was assigned the median value of its interval, for example, 10%–20% = 15%.

For site characterization, the ground vegetation cover was visually estimated in the early growing season (late June/early July) as the average coverage (%) within 3 randomly selected squares of 3x3 m per site. Dwarf shrubs were determined to the species level, while mosses, lichens, graminoids, and forbs were identified to functional group level.

The Luftwebb record for 2000–2014 shows a range in mean annual air temperature across our transects between −1.1 ± 0.2 and 0.3 ± 0.2°C (mean ± *SE*), and the mean annual precipitation ranged from 447 ± 17 to 1,366 ± 34 mm/year. The mean growing season temperature ranged between 9.9 ± 0.3 and 11.3 ± 0.3°C. To capture the smaller scale variation in climate at the site level, the soil temperature was measured with iButtons (Maxim Integrated, San Jose, CA, USA) installed in the topsoil (5–10 cm depth, 1 at the center of each site) from early September 2015 until late August/early September 2017. We used the mean growing season soil temperature as a predictor (averaged between 15/6 and 15/9) to get the most relevant temperature for aboveground processes and to reduce the legacy effects of varying snow‐cover between sites. The soil moisture (volumetric water content, %) was estimated as an average of ~15 measurements per site with a Campbell Hydrosense II moisture sensor (~10 cm depth) (Campbell Scientific Inc., Logan, UT, USA) at every site visit. For statistical analyses, we used the early growing season moisture content (late June/early July), as the topsoil was very dry at some sites in the late season, which might make these measurements less representative as a proxy for root zone water availability.

### 2.3 Chemical analyses

Total C and N concentrations of soils (topsoil: 4 samples; subsoil: 1 composite sample from minimum 3 rings per site) and leaves (composite sample from ~30 to 50 leaves from minimum 7 trees per site) were measured in solid samples (soils: 7 mg, leaves: 5 mg) by Dumas combustion (1,040°C) on an elemental analyzer (Eurovector CN analyser, Redevalle, Italy) after thorough homogenization in a ball mill. Total P content was determined using a FIAstar 5000 flow injection analyzer (FOSS, Hillerød, Denmark) on 25 mg samples, after total dissolution in H_2_SO_4_ with Se. Condensed tannin content was determined in 100 mg leaf material extracted in 5 ml methanol using the vanillin method with catechin as standard, and a Hitachi U 2010 spectrophotometer (Hitachi, Tokyo, Japan).

### 2.4 Estimation of fluxes and resorption

The site fluxes of C, N, and P through insect herbivores (*X*_herb_, g m^−2^ yr^−1^) were estimated as the product of the leaf production (LP, g m^−2^ yr^−1^), proportion of the leaf area lost to herbivory (H), and the elemental proportions in the green leaves collected in the early growing season ([*X*_green_]):

$$X_{\text{herb}}=\text{LP}\times H\times \left[X_{\text{green}}\right].$$

Similarly, the flux of elements through the litter (*X*_litter_, g m^−2^ yr^−1^) was estimated as the product of the leaf production (LP, g m^−2^ yr^−1^) corrected for the proportion lost to herbivory (*H*), and the elemental concentrations in the senesced leaves collected in the late growing season ([*X*_sen_]):

$$X_{\text{litter}\;i}=\left(1‐H_{i}\right)LP_{i}\times {\left[X_{\mathrm{sen}}\right]}_{i}$$

Foliar nutrient resorption (*X*_resorp_, %) was calculated according to van Heerwaarden et al. (2003). Briefly, it was estimated as the difference in nutrient concentrations between the green and the senesced leaves normalized to leaf area, and expressed as a fraction of the green leaf nutrient content. Nutrient resorption needs to be accounted for, when comparing fluxes based partly on green leaves (herbivore deposits), and partly on senesced leaves (leaf litter), as strong resorption amplifies the relative importance of the green leaf component, that is, the herbivory‐mediated flux.

### 2.5 Statistical analyses

Descriptive statistics were expressed as arithmetic means and standard errors (SE). When feasible, errors were propagated to maintain an estimate of variance. We tested correlations between our predictor variables and herbivory levels as well as the annual proportion of elemental fluxes using linear mixed‐effect modeling (‘lmer’ function in ‘lme4’ package, Bates et al., 2015). Confidence intervals (95%) of the effects sizes were constrained with 999 parametric bootstrap simulations, to obtain a conservative estimate of significance that makes no assumptions about degrees of freedom, as this is not trivial to obtain for linear mixed‐effect models (Luke, 2017). Effects were considered significant when the CIs did not overlap zero. The local elevation was included in the models as *Z*‐scores (mean = 0, unit = standard deviations, *SD*) based on the site elevation grouped by transect. This standardisation yields values close to −1 for the lowest site, close to 0 for the middle site, and close to +1 for the upper site. This standardisation was done to correct for the differences in phenology and treeline at similar absolute elevation on north‐ and south‐facing slopes and only look at the effect of relative position above valley bottom. Similarly, regional elevation was transformed to *Z*‐scores centered on the mean elevation of the entire dataset. For herbivory levels, we tested volumetric soil water content, mean growing season soil temperature, annual solar radiation, relative position above the valley bottom, and regional elevation as abiotic predictors, while leaf C:N and CT:C ratios were included as biotic predictors. When testing these as fixed effects, the other variables were classified (4 approximately normally distributed groups per variable) and included as random effects (see full model output at the Figshare link provided in the *Data Availability* section). Further, the transect number was included as a random effect in all models (9 groups). For the annual proportion of elemental fluxes, we tested herbivory level, leaf production, leaf nutrient (N or P) content, and nutrient (N or P) resorption as fixed effects. Nutrient resorption was included, as strong nutrient resorption would decrease the nutrient flux through senesced litter and result in a larger proportion of the annual nutrient fluxes through insect deposits for a given leaf area loss. Variables were transformed when needed to comply with model assumptions and assure similar variance for all variables. All statistical analyses were conducted in R version 3.6.2 (R Core Team, Vienna, Austria).

**Figure 2:**
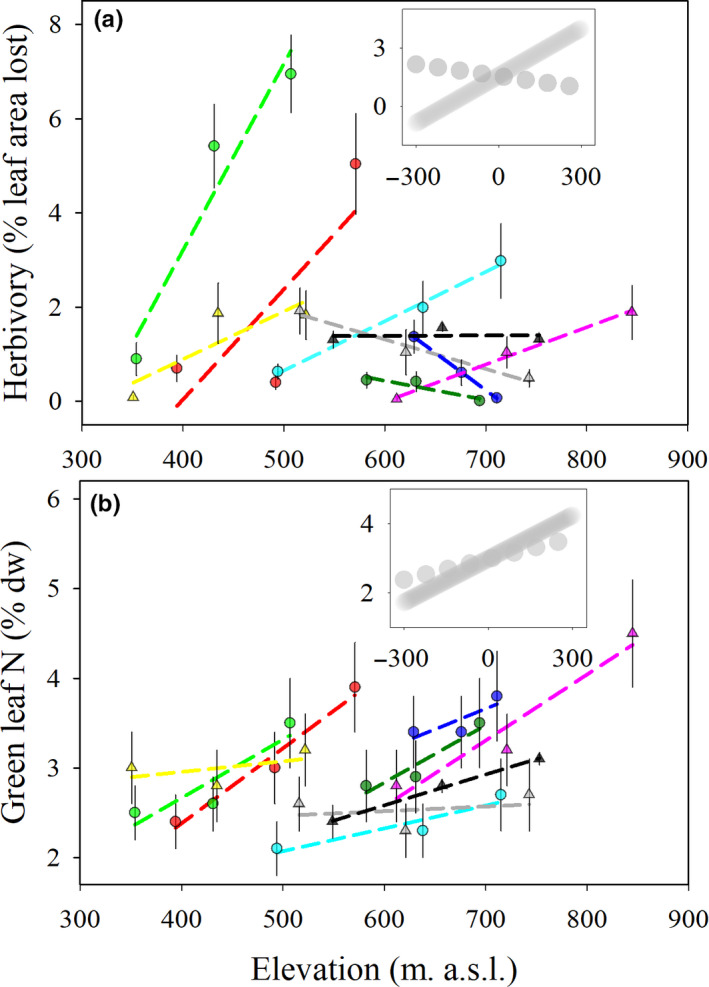
Elevational trend in background insect herbivory (a) and green leaf N content (b). Triangles represent gradients along south‐facing slopes, while circles represent north‐facing gradients. Each color represents an individual elevational gradient (colors correspond to the transect names in Figure 1). Dashed lines show the correlation lines for individual gradients (*n* = 3). Note that these are only plotted to aid the visual interpretation of the trend along each gradient. Error bars show standard errors. In the insets, the dotted line shows the approximated linear trend with regional scale elevation, while the solid line shows the trend with local scale elevation (*n* = 27). These trendlines are also for visual interpretation only, while we refer to the linear mixed‐effect modeling outputs for statistical evaluation of the relationships (Table 2)

To make a visual representation of the relationship between local or regional elevation and herbivory, we plotted correlation lines between BIH and the local elevation (site elevation centered per transect, i.e., mean transect elevation = 0, unit: m. above sea level) and the regional elevation (site elevation centered on the mean elevation of the entire dataset, i.e., mean elevation = 0, unit: m. above sea level) (as insets in Figure 2). However, these lines, as well as the correlation lines in the main panels (Figure 2) are for visual guidance only. For effect sizes and evaluation of significance, we refer to the linear mixed‐effect modeling results (Table 2a). We included green leaf N content (% of mass) plotted against elevation to check the design, as we expected a more consistent positive relationship between leaf N and elevation, while the expectation to foliar loss to herbivores was less straight forward (see introduction).

## 3 RESULTS

### 3.1 Insect herbivory and elemental fluxes

The mean leaf area lost to BIH across all sites was ~1.6%, while ~10% of the leaves sampled across all sites were subjected to some degree of insect herbivory (Table 1). The proportional leaf area lost to herbivory was positively correlated with the relative position above the valley bottom (CI = 0.24; 0.72) and leaf N content (negative correlation with C:N, CI = −0.21; −0.00), yet showed no significant link to annual solar radiation, temperature, regional elevation, or leaf content of condensed tannins (Table 2a).

**Table 1:** Ecological characteristics

Variable	Unit	Mean	Min	Max
Leaf production				
LAI	m^2^ leaf m^−2^ ground	**0.89 ± 0.1**	0.05 ± 0.02	1.91 ± 0.25
SLA	m^2^/g dw	**0.019 ± 0.0006**	0.015 ± 0.0008	0.027 ± 0.001
Leaf production	g dw/m^2^ ground	**47 ± 5**	2.9 ± 1.07	104 ± 13.8
Green leaves				
C	%dw	**43 ± 0.2**	41 ± 3	46 ± 3
N	%dw	**3.0 ± 0.11**	2.1 ± 0.3	4.5 ± 0.6
P	%dw	**0.29 ± 0.01**	0.17 ± 0.03	0.43 ± 0.07
CT	%dw	**0.52 ± 0.13**	0.004 ± 0.002	1.9 ± 1.2
C:N		**15 ± 0.5**	9 ± 1	21 ± 2
C:P		**160 ± 7.2**	95 ± 11	248 ± 27
CT:C	‰	**12 ± 41**	0.09 ± 0.06	45 ± 29
Senesced leaves				
C	%dw	**44 ± 0.3**	43 ± 1	47 ± 1
N	%dw	**1.2 ± 0.06**	0.8 ± 0.1	1.9 ± 0.3
P	%dw	**0.18 ± 0.008**	0.1 ± 0.02	0.25 ± 0.06
C:N		**38 ± 1.8**	23 ± 4	58 ± 9.8
C:P		**254 ± 12**	172 ± 41	444 ± 107
Resorption				
N	%	**56 ± 2.8**	23 ± 5	81 ± 18
P	%	**30 ± 4.1**	−11 ± 4	81 ± 26
Litter input				
C	g/m^2^	**21 ± 2.2**	1.3 ± 0.47	45 ± 6.1
N	g/m^2^	**0.6 ± 0.08**	0.03 ± 0.012	1.6 ± 0.31
P	g/m^2^	**0.09 ± 0.011**	0.006 ± 0.0023	0.24 ± 0.032
Herbivory level				
prop. of leaf area	%	**1.6 ± 0.33**	0.01 ± 0.012	7 ± 0.82
prop. of leaves damaged	%	**10 ± 1.6**	0.48 ± 0.476	42 ± 5.34
Insect deposits				
C	mg/m^2^	**250 ± 48**	3 ± 3	1,030 ± 340
N	mg/m^2^	**17 ± 3.3**	0.3 ± 0.26	60 ± 21
P	mg/m^2^	**1.7 ± 0.32**	0.2 ± 0.023	6.2 ± 2.0
prop. of annual C	%	**1.5 ± 0.3**	0.01 ± 0.012	6.3 ± 3.7
prop. of annual N	%	**3.6 ± 0.7**	0.03 ± 0.028	14.1 ± 7.3
prop. of annual P	%	**2.4 ± 0.5**	0.03 ± 0.031	9 ± 5.9

Note

All variables are presented as an overall mean for all sites (MEAN), as well as the minimum (MIN) and maximum (MAX) values to show the variation across sites. Uncertainties show standard errors for all samples (MEAN, *n* = 27), and per site (MIN, MAX, *n* = 3). LAI: leaf area index, SLA: Specific leaf area. CT: Condensed tannins. Note that the insect deposits are in [mg/m^2^] while litter inputs are in [g/m^2^]. Insect deposits are also presented in proportion to the total input (leaf litter plus herbivore deposits).

**Table 2:** Linear mixed‐effect modeling results for variables explaining variation in herbivory level (a, % of leaf area lost, sqrt transformed) and green leaf N content (b, % dry mass)

(a)				
Variables	Unit	Coeff.	CI, lo	CI, up
Elevation	Z‐score (all)	−0.12	−0.48	0.22
**Relative position (local elevation)**	**Z‐score (per transect)**	**0.48**	**0.24**	**0.72**
Annual solar radiation	MWH m^−2^	3.75	−0.32	7.59
Mean growing season soil temperature	°C	0.10	−0.15	0.33
Volumetric soil water content (early GS)	%	0.02	−0.02	0.05
**Green leaf C:*N***		**−0.10**	**−0.21**	**−0.00**
Green leaf CT:C_log_		−0.05	−0.44	0.34

(b)				
Variables	Unit	Coeff.	CI, lo	CI, up
**Elevation**	**Z‐score (all)**	**0.39**	**0.23**	**0.55**
**Relative position (local elevation)**	**Z‐score (per transect)**	**0.41**	**0.26**	**0.57**
Annual solar radiation	MWH m^−2^	0.12	−2.26	2.38
Mean growing season soil temperature	°C	−0.15	−0.31	0.02
Volumetric soil water content (early GS)	%	0.02	−0.00	0.05
Leaf production	g dw/m^2^ ground	−0.00	−0.01	0.00

Note

Variables with significant predictive power (CI do not overlap zero) are highlighted in bold. The coefficients (Coeff.) show the effect size and are included primarily to show the direction of change. The upper (CI, up) and lower (CI, lo) 95% confidence intervals of the effect size (Coeff.) after 999 parametric bootstrap simulations were used to evaluate significance. Note that the site elevation was transformed to Z‐scores, partly to make the units comparable to local elevation, and partly to make the variance more similar to the other variables.

The overall mean elemental canopy loss to herbivores was estimated to ~250 mg C, ~17 mg N, and ~2 mg P m^−2^ yr^−1^ (Table 1). The mean leaf production across sites was ~47 g dry matter m^−2^ yr^−1^, corresponding to ~21 g C, ~0.6 g N, and ~0.09 g P m^−2^ yr^−1^ (Table 1). The estimated proportion of nutrients resorbed from the birch leaves toward the end of the season was ~56% for N and ~30% for P (Table 1). The resulting proportion of the annual flux from the canopy through insect deposits make up ~1.5% of the total C flux (litter + herbivore input), while the proportion of N and P were slightly higher at ~3.6 and ~2.4%, respectively, due to foliar nutrient resorption during senescence. The annual proportion of canopy‐to‐soil N and P fluxes caused by herbivory were positively correlated with herbivory level (N: CI = 0.143; 0.161 and P: CI = 0.112; 0.122) and resorption (N: CI = 0.001; 0.003 and P: CI = 0.000; 0.001, Table S2). Moreover, the proportional insect‐mediated flux of N was negatively correlated with leaf production (N: CI = −0.001; −0.000). There was no significant link to leaf N or P concentrations (Table S2).

### 3.2 Consistency between local and regional elevational trends

Our design was set up to examine the consistency between local and regional elevational trends in BIH (inset in Figure 2a). Yet, while the leaf area lost to herbivory increased with local elevation along each transect (visualized by solid line in inset Figure 2, CI = 0.24; 0.72, Table 2a), it showed an insignificant neutral‐to‐negative relationship with regional elevation (visualized by dotted line in inset Figure 2, CI = −0.48; 0.22, Table 2a). In contrast, the green leaf N content showed a similar increase with elevation along both the regional (CI = 0.23; 0.55) and local (CI = 0.26; 0.57) gradients (inset in Figure 2b, Table 2b).

## 4 DISCUSSION

### 4.1 Herbivory‐mediated canopy‐to‐soil fluxes and their controls

Our results showed that foliar loss to BIH constitutes a minor fraction of the annual soil input of organic matter from the birch canopies, in line with our hypothesis (H_1_). The mean leaf area loss of ~1.6% is similar to the estimates of leaf area loss to insect herbivory in dwarf shrub tundra (Barrio et al., 2017). Yet, the loss is considerably lower than in ecosystems at lower latitudes (Galmán et al., 2018; Hunter et al., 2003), and the global average of 7.55% of the leaf area, showing a dome‐shaped latitudinal distribution, that is, the highest rates at temperate latitudes with a sharp decline toward the polar regions (Kozlov, et al., 2015). This distribution emphasizes the potential increase in BIH in cold ecosystems with climate warming. Despite the relatively short recurrence interval of insect population peaks of ~10 years (Jepsen et al., 2008), major outbreaks only return to the same site every ~50–100 years (Tenow & Bylund, 2000). Thus, the relatively small contributions to fluxes at low insect densities may cumulatively recycle similar or larger amounts of elements than the major outbreaks, where most of the leaf area is lost, but it is much less disruptive to the ecosystem. Further, as background densities may increase with future climate warming (Galmán et al., 2018; Kozlov, et al., 2015), BIH may increase in overall importance for nutrient cycling. The variation in insect herbivory level across our sites was considerable, with leaf area loss at some sites of up to 7%. When the nutrient resorption during leaf senescence (~55% N, ~30% P) was accounted for, the average herbivore‐mediated fraction of the annual soil input was slightly higher than for C (~3.5% N and ~2.5% P), yet still a relatively small contribution. Nonetheless, at the sites with the highest herbivory levels, this corresponded to ~14% and ~9% of annual N and P fluxes, respectively. These estimates of herbivory‐mediated fluxes may be in the low end, as our estimated mean N resorption efficiency of 55% was lower than what has previously been found in these forests (~60%–70%, Freschet et al., 2010; Nordell & Karlsson, 1995). This suggests an even larger gap between the substrate quality of litter and insect deposits. The higher substrate quality (availability to soil decomposer microbes) of insect deposits compared to litter (Kristensen et al., 2018) may trigger increased soil N and C‐turnover early in the season. Yet, these belowground responses to BIH are most likely within the ranges of what the soil biota can take up and keep within the ecosystem, and so are unlikely to cause nutrient losses, in contrast to outbreaks which can cause substantial nutrient losses from the soil (Hunter 2001, Kristensen et al., 2020; Lovett et al., 2002).

The positive relationship between herbivory and leaf N concentration in the green leaves was in line with our expectations (H_2_), suggesting that leaves of higher nutritive quality suffer greater herbivore damage, with no apparent effect of foliar condensed tannin concentration. Moreover, the strong negative relationship between leaf N content and condensed tannins (linear regression w. CT:C log‐transformed, *p* < .001) corroborates the theory of Haukioja (2003) predicting that it is only beneficial for the plants to increase the content of defense compounds when the nutritive quality of the leaves is low. Only under such conditions, the insects will eat large enough amounts of the leaves to encounter a negative effect from the tannins. We note that our measurements of leaf condensed tannin content were quite low compared to some other literature observations from similar trees and ecosystems (Paaso et al., 2017; Stark et al., 2007). This may be partly due to large variation between sampling years, genotypes, and responses to other regulators than herbivory, such as soil nutrient content and photochemical conditions (Madritch & Lindroth, 2015; Rubert‐Nason et al., 2015).

The significant increase in insect herbivory with relative position above the valley bottom, that is, toward the treeline, was in line with our expectations (H_3_) derived from the patterns found for outbreaks (Hagen et al., 2007), and previous indications from studies of nonoutbreak conditions in subarctic Finland (Virtanen & Neuvonen, 1999). Yet, this is, to our knowledge, the first time a significant effect has been shown for background herbivore densities. Despite the overall increase in BIH with local elevation, the relationship is not consistently positive across all gradients. In fact, there seems to be a systematic decrease in the slope along individual gradients (dashed lines in Figure 2) with increasing regional elevation, that is, the increase in BIH with local elevation goes from being positive at low regional elevation, to being neutral to negative at higher regional elevation. This transition is clearest for the north‐facing slopes (circles, Figure 2), so one could speculate that when solar energy is abundant (south‐facing slopes, triangles, Figure 2) BIH is controlled by mechanisms that are to a lesser extent correlated with elevation. Transects at higher regional elevation show the lowest average BIH levels, which may be due to higher exposure to extremely low winter temperatures at these high elevation transects located in relatively deep valleys close to the regional tree line (Figure 1), hence closer to the absolute range limit of the insects. Nonetheless, the mechanisms remain unclear. The proposed drivers of the patterns—higher parasitism in valley bottoms during the summer and eggs being killed by extremely cold air masses creeping into valley bottoms during the winter (Virtanen & Neuvonen, 1999)—are confounded by the consistent increase in leaf N content with local elevation. Yet, if foliar chemistry was the paramount driver of BIH across scales, the N concentration should show a neutral‐negative trend with elevation at the regional scale. In contrast, the leaf N content also increase with elevation at the regional scale, although the relationship is slightly weaker than at the local scale as should be expected (Read et al., 2014). Thus, abiotic drivers, for example, climate, are likely moderating the biotic relationships at the regional scale.

The local increase in herbivory with elevation found here contrasts with the more general decrease with elevation along individual transects across the World's woody species (Galmán et al., 2018). This suggests that the proposed responsible mechanisms in subarctic mountain birch landscapes may only apply to high‐latitude systems. Thus, our study serves as a useful example of how global generalisations may lead to wrong assumptions in certain ecosystems highly important for the global climate. Nonetheless, as single‐year data are not always representative of general patterns (Kozlov et al., 2013; Zvereva & Kozlov, 2019) these data should be interpreted with caution. Historical data have shown that climatic drivers are important for population dynamics and ranges in subarctic birch forests (Jepsen et al., 2008), but the underlying mechanisms and consequently predictions remain challenging (Jepsen et al., 2008, 2009; Vindstad et al., 2019).

### 4.2 Comparison to other ecosystem fluxes and theoretical implications

The contribution by insect herbivores at background densities through leaf consumption was relatively small, as we expected (H_1_). The annual canopy litter inputs to soils were estimated to ~20 g C, ~0.5 g N, and ~0.1 g P/m^2^ on average (Table 1). This is comparable to other literature estimates in terms of biomass (Dahlberg et al., 2004; Kjelvik & Kärenlampi, 1975), but 1–2 orders of magnitude more than the flux of the same elements through insect herbivores at background densities (Table 1).

Relevant external nutrient inputs to the subarctic birch forests are atmospheric N deposition, which was estimated to ~0.05–0.1 g N m^−2^ yr^−1^ in the area for the period 2013–2015 (Alpfjord & Andersson, 2017), biological N fixation, which has been estimated to be 0.1–0.5 g N m^−2^ yr^−1^ (Jonasson & Michelsen, 1996; Rousk & Michelsen, 2017; Rousk et al., 2016) and P from mineral weathering, which has been estimated to contribute ~0.01 g P m^−2^ yr^−1^ (Akselsson et al., 2008). This shows that the external inputs of N were comparable to our estimates of what was recycled through the litter, but 1–2 orders of magnitude higher than the contribution by insect herbivores at background densities. The external input of P to the soil via weathering was about an order of magnitude lower than the annual litter input, which reinforces evidence that internal P recycling from organic matter is crucial for plant production in these high‐latitude systems (Sundqvist et al., 2014). Yet, the input of P through weathering was still 1–2 orders of magnitudes higher than the contribution to the canopy‐to‐soil flux through insect herbivores. It is, however, important to emphasize that only a small fraction of the nutrients transferred through litter (slow cycle) ends up as part of the immediately available pool of soil nutrients in boreal ecosystems (Jonsson & Wardle, 2008; Metcalfe et al., 2016). So in terms of relieving nutrient limitations to plant growth, smaller labile inputs (fast cycle) from insects may be equally or more important.

Leaf consumption is not the only way insect herbivores influence the partitioning between fast‐ and slow‐cycle pathways through the decomposer foodweb (Hunter et al., 2012; Schowalter, 2016). Premature abscission of green leaves can increase the defoliation considerably beyond the level of leaf consumption (Schowalter et al., 2011; Zvereva & Kozlov, 2014), although plants invaded by leaf defoliators show a lower overall abscission rate compared to other guilds, that is, leaf miners and gallers (Zvereva & Kozlov, 2014). Further, insect herbivory increases the leaching of labile organic matter and nutrients in throughfall solution making considerable contributions to soil nutrient availability in some forest ecosystems (le‐Mellec et al., 2011; Schowalter, 2016; Schowalter et al., 2011). These increased soil surface inputs caused by insect herbivores further enhance the fast‐cycle pathway, although we did not quantify them here.

Studies from grassland systems have shown that insect herbivory increases belowground allocation of photosynthates by their hosts, possibly to boost their nutrient uptake (Belovsky & Slade, 2000; Hamilton et al., 2008). Yet, in forest ecosystems, belowground C allocation by plants is reduced by aboveground insect herbivory (Frost & Hunter, 2008a; Kristensen et al., 2020), which represents a reduction in fast‐cycle processes. Moreover, the immediate induction in chemical plant defenses (Fürstenberg‐Hägg et al., 2013; Halitschke et al., 2008; Haukioja, 2005; Kessler et al., 2004) increase the decomposition time of the resulting litter (Chomel et al., 2016), although it is not clear to what extent these effects may be transient (Frost & Hunter, 2008b). Nonetheless, existing frameworks predict that continuous exposure of plant communities to generalist herbivores may eventually drive a shift toward more resistant, less palatable assemblies, which may further enhance the slow‐cycle pathway in the long term (Bardgett & Wardle, 2010; Wardle et al., 2004).

Overall, while insect herbivores may primarily enhance fast‐cycle pathways in fertile grassland systems, where compensatory growth responses are common (Hamilton et al., 2008), the picture is more mixed in forest ecosystems. Increased inputs of insect deposits to the soil surface increase fast‐cycle processes. In contrast, decreased allocation of photosynthates to belowground plant parts and root‐associated microbes, as well as the decreased litter availability to decomposers, enhances slow‐cycle processes. Thus, to fully understand the role of herbivores in global change research, we need to take such biome dependencies, and the interactions between different herbivore functional groups (e.g., Risch et al., 2018) into account. Herbivory on mountain birch in our study area is strongly dominated by leaf‐eating geometrid moths at present (Jepsen et al., 2008; Tenow & Bylund, 2000), although other insect herbivores are present (Kozlov et al., 2017). Thus, we believe our flux estimates are useful to understand current ecosystem functioning. However, leaf miners, gallers, phloem feeders, and wood borers are important disturbance agents in other forest ecosystems (Kirichenko et al., 2019; Kozlov, et al., 2015; Kurz et al., 2008; Schowalter, 2016; Sugiura, 2010) and may therefore become more important under warmer climate conditions in subarctic Fennoscandia as well.

### 4.3 Inferences from studies along natural elevational gradients

Despite the contrasting elevational trends along individual transects in our study (increasing) and the general global trend (decreasing) found by Galmán et al. (2018), our results confirm that the variation in insect herbivory with elevation along individual transects cannot be explained entirely by traditional climate variables (Galmán et al., 2018). Inconsistent relationships between elevation and ecological characteristics, when comparing multiple individual gradients and regional scale variation, have been shown for a multitude of above‐ and belowground variables (Read et al., 2014; Sundqvist et al., 2013). While adding ecological context, for example, field‐layer vegetation (Mayor et al. 2017) or grazer/browser presence (Bernes et al., 2015; Vowles et al., 2017), may sometimes be sufficient to explain unexpected trends, the complex geometry of mountainous landscapes poses some challenges to the simplistic, implicit assumption of universality in the relationship between elevation and abiotic variables, such as temperature (Körner, 2007). Further, we often implicitly assume that the driver and response variable change at similar rates, that is, are in a steady state, but this is for instance rarely the case in studies of plant community compositional responses to climate change along natural gradients (Damgaard, 2019; Hagedorn et al., 2019). Some of the discrepancies in observed elevation patterns likely reflect difference in variables, methods, and variation in the extent to which confounding factors obscure shifts solely related to temperature change with elevation. Our study provides a useful test of the power of elevation gradients by recording the same suite of variables using the same methods across multiple gradients within the same ecosystem in a rather constrained geographical area. While the relationship with elevation was rather consistent across scales for some variables (leaf N content), we found considerable variation in elevation trends in others (leaf herbivory level). Yet, identifying useful moderator variables (e.g., solar radiation) to account for these scale‐dependent differences in trends might be a way to allow for quick integration of space‐for‐time substitution data from different geographical contexts into ecosystem models. This could improve predictions of important long‐term responses to climate change (Dunne et al., 2004; Elmendorf et al., 2015), until sufficient understanding of the underlying mechanisms emerges. Thus, to understand the effect of elevation at a broader scale, we need both local elevational gradient data and regional moderator variables. For example, the contrasting relationship between elevation and BIH in the Subarctic, shown in our study, and the trend at lower latitudes, may be incorporated into models by using latitude as a moderator variable. This approach needs further confirmation, but we show that setting up multiple elevation gradients in the same biome within a rather constrained area yield useful data for such assessments.

## 5 CONCLUSION

We showed that the leaf area loss due to background insect herbivory (~1.6%) in subarctic birch forests was comparable to what was previously found in dwarf shrub tundra, yet lower than ecosystems at lower latitudes, and an increase with climate warming should therefore be anticipated. The nutrient fluxes from canopy to the soil associated with current background herbivore intensities were 1–2 orders of magnitude lower than the fluxes through senesced litter and soil input from external sources. The variation in background insect herbivory rates was substantial, however, and showed an increase with elevation at the local scale. There was no link to variation in overall elevation, temperature, humidity, nor annual solar radiation. This is in line with previous reviews suggesting that the most commonly recorded climatic driver variables, temperature and humidity, are not always sufficient to predict variation in insect herbivory and other ecological processes in mountainous landscapes. We speculate that accounting for context dependencies by introducing moderator variables in ecosystem models, for example, latitude or biome, may be a fast way forward to allow better integration of data from elevational gradients, until we have sufficient understanding of the discrepancies between biotic–abiotic relationships in space‐for‐time substitution studies at different scales.

## CONFLICT OF INTEREST

The authors declare no conflicts of interest.

## AUTHOR CONTRIBUTION

**Jeppe Aagaard Kristensen:** Conceptualization (equal); Data curation (lead); Formal analysis (lead); Funding acquisition (supporting); Investigation (lead); Methodology (equal); Project administration (equal); Resources (equal); Visualization (lead); Writing‐original draft (lead). **Anders Michelsen:** Formal analysis (supporting); Methodology (supporting); Resources (equal); Supervision (supporting); Writing‐review & editing (supporting). **Dan Metcalfe:** Conceptualization (equal); Data curation (supporting); Funding acquisition (lead); Investigation (supporting); Methodology (equal); Project administration (equal); Resources (supporting); Supervision (lead); Visualization (supporting); Writing‐review & editing (equal).

## Supporting information

Supplementary Material

## Data Availability

The data are available at Figshare with the following https://doi.org/10.6084/m9.figshare.12840134. The full linear mixed‐effect regression outputs are available at the same repository with https://doi.org/10.6084/m9.figshare.12840320.
