# Supplementary material for: Background insect herbivory increases with local elevation but makes minor contribution to element cycling along natural gradients in the Subarctic
Source: Ecol Evol. 2020 Sep 21;10(20):11684–98. doi: 10.1002/ece3.6803 (PMC7593201; doi:10.1002/ece3.6803)
Supplement: Supplementary file 1 — Supplementary Material [file ECE3-10-11684-s001.docx]

Supplementary information for

**Background insect herbivory increases with local elevation but makes minor contribution to element cycling along natural gradients in the Subarctic.**

Extended materials and methods

Table S1: Site characteristics

Table S2: Linear mixed effect regression of the annual proportion of N and P fluxes through insect deposits

LMER_output: Excel sheet with model output from the linear mixed effect regressions available at Figshare with DOI: 10.6084/m9.figshare.12840320

Data: Datasheet available at Figshare with DOI: 10.6084/m9.figshare.12840134

**Extended materials and methods**

*Soil solution chemistry*

Three resin capsules (UNIBEST International, Walla Walla, WA, USA) were installed in the topsoil (5–10 cm) over one entire year (extracted in August 2016) to estimate inorganic soil solution nutrient content at each site. According to the provider, this data should be seen as an integrated equilibrium of the soil solution chemical composition over the last ~5-7 weeks before extraction. We are aware that this is not the same season as the other sampling was performed (2017), but we did not expect the relative difference between sites to vary substantially between years, as the soil solution chemistry usually show much larger spatial and seasonal variation than interannual heterogeneity if no major disturbances occur (e.g. Manderscheid and Matzner 1995). Chemicals absorbed on the resin capsules were analysed by the provider (UNIBEST International, Walla Walla, WA, USA). Briefly, ammonium and nitrate concentrations were analysed on a flow injection analyser (FIAlab-2500, FIAlab Instruments, Inc., Seattle, WA, USA), while all other elements were analysed using inductively coupled plasma optical emission spectroscopy (ICP-OES, Perkin-Elmer, Waltham, MA, USA) after extraction with 50 ml 2 M HCl. Here, we only present data for P and cumulative base cations (Ca^2+^, Mg^2+^, K^+^, Na^+^), which is another essential group of plant nutrients, to give a sense of the general nutrient status of the soil.

**References**

Manderscheid B, Matzner E. 1995. Spatial and temporal variation of soil solution chemistry and ion fluxes through the soil in a mature Norway Spruce (Picea abies (L.) Karst.) stand. *Biogeochemistry* **30**: 99-114.

**TABLE S1: Site characteristics.** All variables are presented as an overall mean for all sites (MEAN), as well as the minimum (MIN) and maximum (MAX) values to show the variation across sites. Uncertainties show standard errors for all samples (MEAN), and per site (MIN, MAX). MAT: Mean annual temperature, MGST: Mean growing season temperature, MAP: Mean annual precipitation.

| **VARIABLE** | | **UNIT** | **MEAN** | MIN | MAX |
| --- | --- | --- | --- | --- | --- |
|  |  |  |  |  |  |
| Air climate (2000-2014) (per transect) | MAT | °C | **-0.3±0.2** | -1.1±0.2 | 0.3±0.2 |
|  | MGST (Jun-Aug) | °C | **10.7±0.9** | 9.9±0.3 | 11.3±0.3 |
|  | MAP | mm year^-1^ | **887±84** | 447±17 | 1366±34 |
|  | Winter precip (Oct-Apr) | mm winter^-1^ | **441±59** | 173±9 | 746±27 |
|  |  |  |  |  |  |
| Soil climate | MAT | °C | **2.5±0.01** | 1.7±0.06 | 4.5±0.2 |
|  | MGST (15/6-15/9) | °C | **8.4±0.01** | 6.7±0.1 | 11.1±0.2 |
|  | Moisture (GS start 2017) | %vol | **19.3±0.5** | 11±1.1 | 38±2.5 |
|  | Moisture (GS end 2017) | %vol | **22±0.4** | 16±1.1 | 31±3.1 |
|  |  |  |  |  |  |
| Dissolved inorganic nutrients | Total inorganic N | µg capsule^-1^ | **10.5±2.2** | 1.07±0.55 | 77.3±28.8 |
|  | NH_4_ | µg capsule^-1^ | **7.2±1.39** | 0.18±0.08 | 50±22.8 |
|  | NO_3_ | µg capsule^-1^ | **3.3±1.36** | 0.74±0.49 | 40.6±25 |
|  | P | µg capsule^-1^ | **10.4±0.92** | 1.68±0.7 | 26.7±10.3 |
|  | Base cations | µg capsule^-1^ | **199±17** | 52±2 | 486±14 |
|  | TIN:P |  | **1.6±0.26** | 0.06±0.03 | 6.4±1.19 |
|  |  |  |  |  |  |
| Forest floor (organic soil) | Depth | cm | **5.9±2.7** | 3.3±0.4 | 11.7±1.3 |
|  | Bulk density | kg m^-3^ | **133±69** | 65±6 | 249±25 |
|  | C | %dw | **29±1.1** | 15±3.9 | 38±2.9 |
|  | N | %dw | **1.2±0.06** | 0.7±0.11 | 1.7±0.1 |
|  | P | %dw | **0.13±0.01** | 0.08±0.01 | 0.23±0.03 |
|  | C:N |  | **25±0.7** | 19±0.7 | 31±1.4 |
|  | C:P |  | **253±20** | 97±40 | 439±68 |
|  | pH |  | **4.6±0.06** | 4.1±0.1 | 5.3±0.31 |
|  |  |  |  |  |  |
| Mineral soil | Depth | cm | **14±16** | 1±0.5 | 54±7 |
|  | Bulk density | kg m^-3^ | **1183±237** | 938±38 | 1568±63 |
|  | C | %dw | **1.4±0.14** | 0.2±0.1 | 3.2±1.1 |
|  | N | %dw | **0.07±0.01** | 0.02±0 | 0.16±0.04 |
|  | P | %dw | **0.06±0.01** | 0.02±0.01 | 0.16±0.05 |
|  | C:N |  | **21±0.8** | 13±6 | 31±14 |
|  | C:P |  | **32±4.7** | 3±2 | 83±39 |
|  | pH |  | **5.1±0.1** | 4.6±0.2 | 5.7±0.2 |
|  | Clay (<2µm) | %dw | **5±1.3** | 0.3±na | 25±na |
|  | Silt (2-63µm) | %dw | **22±3.7** | 6±na | 66±na |
|  | Sand (63-2000µm) | %dw | **74±4.9** | 12±na | 94±na |
|  |  |  |  |  |  |
| Ground vegetation | Herbs | % of area | **34±6** | 0.7±0 | 89±23 |
|  | Mosses and lichens | % of area | **19±3** | 0.3±0 | 61±25 |
|  | Dwarf shrubs | % of area | **51±5** | 0±0 | 100±34 |

**TABLE S2: Linear mixed effect modelling results for variables explaining variation in insect herbivory contributions to annual canopy-to-soil fluxes of N (a) and P (b) (% of annual flux, sqrt transformed).** Variables with significant predictive power (CIs do not overlap zero) are highlighted in bold. The coefficients (Coeff.) show the effect size and are included primarily to show the direction of change. The upper (CI, up) and lower (CI, lo) 95 % confidence intervals of the effect size (Coeff.) after 999 parametric bootstrap simulations are used to evaluate significance. Note that the site elevation was transformed to Z-scores, partly to make the units comparable to local elevation, and partly to make the variance more similar to the other variables.

(a)

| Variables | Unit | Coeff. | CI, lo | CI, up |
| --- | --- | --- | --- | --- |
| **Leaf productivity** | **g m^-2^** | **-0.0007** | **-0.0012** | **-0.0001** |
| Leaf C:N |  | -0.0007 | -0.0075 | 0.0059 |
| **Leaf N-resorption** | **% of green leaf N** | **0.0015** | **0.0006** | **0.0025** |
| **Herbivory level_sqrt_** | **% of leaf area** | **0.152** | **0.143** | **0.161** |

(b)

| Variables | Unit | Coeff. | CI, lo | CI, up |
| --- | --- | --- | --- | --- |
| Leaf productivity | g m^-2^ | -0.0003 | -0.0009 | 0.0002 |
| Leaf C:P |  | -0.0001 | -0.0005 | 0.0003 |
| **Leaf P-resorption** | **% of green leaf P** | **0.0005** | **0.000** | **0.001** |
| **Herbivory level_sqrt_** | **% of leaf area** | **0.117** | **0.112** | **0.122** |
